# rs657075 (*CSF2*) Is Associated with the Disease Phenotype (BAS-G) of Ankylosing Spondylitis

**DOI:** 10.3390/ijms18010083

**Published:** 2017-01-03

**Authors:** Wei-Chiao Chen, James Cheng-Chung Wei, Hsing-Fang Lu, Henry Sung-Ching Wong, Peng Yeong Woon, Yu-Wen Hsu, Jin-Ding Huang, Wei-Chiao Chang

**Affiliations:** 1 Institude of Clinical Pharmacy and Pharmaceutical Sciences, College of Medicine, National Cheng Kung University, Tainan 70101, Taiwan; doggyenjoying@yahoo.com.tw; 2 Division of Allergy, Immunology and Rheumatology, Chung Shan Medical University Hospital, Taichung 40201, Taiwan; wei3228@gmail.com; 3 Institute of Medicine, Chung Shan Medical University, Taichung 40201, Taiwan; 4 Institute of Integrative Medicine, China Medical University, Taichung 40201, Taiwan; 5 Department of Clinical Pharmacy, School of Pharmacy, Taipei Medical University, Taipei 11014, Taiwan; shefunlu@gmail.com (H.-F.L.); miningyue@gmail.com (H.S.-C.W.); 6 Master Program for Clinical Pharmacogenomics and Pharmacoproteomics, School of Pharmacy, Taipei Medical University, Taipei 11014, Taiwan; 7 Department of Molecular Biology and Human Genetics, Tzu Chi University, Hualien 97004, Taiwan; woon07@mail.tcu.edu.tw; 8 The Ph.D. Program for Translational Medicine, College of Medical Science and Technology, Taipei Medical University and Academia Sinica, Taipei 11031, Taiwan; fish770426@hotmail.com; 9 Center for Biomarkers and Biotech Drugs, Kaohsiung Medical University, Kaohsiung 80708, Taiwan; 10 Department of Pharmacy, Taipei Medical University-Wanfang Hospital, Taipei 116, Taiwan

**Keywords:** ankylosing spondylitis, *CSF2*, rs657075, Bath AS Global (BAS-G), single nucleotide polymorphism

## Abstract

Ankylosing spondylitis (AS) is a systemic autoimmune disease mainly affecting the lumbar spine and sacroiliac joints, and exhibits peripheral inflammatory arthropathy. More than 25 loci have been identified as associated with AS. Because both AS and rheumatoid arthritis (RA) are autoimmune diseases that may share some common genetic factors, we therefore examined if the newly identified RA genetic polymorphisms were associated with AS in a Taiwanese population. In this study, we enrolled 475 AS patients and 11,301 healthy subjects from a Taiwanese biobank as controls. Although none of single-nucleotide polymorphisms (SNPs) were associated with the susceptibility to AS, the AS disease index Bath AS Global (BAS-G) clinical phenotype was observed as significantly correlated to the AA genotype of rs657075 (*CSF2*). The significance remains after gender/age/disease duration adjustment and after group categorization by *human leukocyte antigen-B 27* (*HLA-B27*) genotype. We further investigated the possible functions of rs657075 through bioinformatics approaches. Results revealed that polymorphism of rs657075 is able to influence the expression of acyl-CoA synthetase long-chain family member 6 (*ACSL6*). In conclusion, our study indicated that rs657075 (*CSF2*) is strongly associated with the AS disease index Bath AS Global (BAS-G) clinical phenotype.

## 1. Introduction

Ankylosing spondylitis (AS) is a systemic autoimmune disease, which is characterized by inflammation of the lumbar spine and sacroiliac joints, peripheral inflammatory arthropathy, and the absence of rheumatoid factor [1,2]. The disease predominantly strikes men between the age of 20 and 40 years, in their peak productive years, leading to significant loss of work productivity and a decreased quality of life [3]. Family and twin studies indicated that genetic factors contribute to over 90% to the overall AS susceptibility [4,5]. *Human leukocyte antigen (HLA)-B 27* has been known as the major AS-susceptibility gene for more than 40 years [6], but *HLA-B27* accounts for only 16% of the genetic variability in AS [7]. The other *HLA-B* allele operative in AS susceptibility is *HLA-B60*, which was identified in studies of US and UK patients with AS [8,9]. In addition, *HLA-B60* and *HLA-B61* were associated with AS in *HLA-B27*-negative patients in Taiwan [10]. The susceptibility to AS can be enhanced when combining different patterns of *HLA-B60* and *HLA-B27* genotypes in Dutch and Taiwanese populations, implying genetic interaction mechanisms that may contribute to AS risk among two genes [11,12]. The *interleukin (IL)-1* and *IL-23R* genes were also proven to be important in the pathogenesis of AS [13,14]. Genes involved in regulating peripheral tolerance were found have a combined effect on the development of AS, such as the *PD-1* G-536A/*PD-L1* A8923C/*PD-L2* C47103T [15] or *PTPN22* G-1123C/*CTLA-4* A49G [16], and the *CTLA-4* +49A/G genotype associated with circulatory C-reactive protein (CRP) level [17]. Our previous studies reported that genetic polymorphisms of *ORAI1* (rs12313273 and rs7135617) and *STIM1* (rs3750996) were associated with the pathogenesis of *HLA-B27*-positive AS patients [18,19].

Additionally, many non-major histocompatibility complex (MHC) regions were found to be significantly associated with AS in genome-wide association studies (GWASs) [20,21,22]. Twelve loci were previously confirmed to be associated with AS in Europeans [20,21,23], 2 loci were recently reported in Han Chinese [22], and an additional 13 new loci were identified in a recent global GWAS, bringing the total AS-associated loci to 43 [24]. Two studies confirmed the findings of previous AS studies that *ERAP1* and rs10865331 are risk factors for AS susceptibility [25,26]. However, some susceptibility loci, such as *EDIL3*, *HAPLN1*, and *ANO6*, discovered in a Han Chinese GWAS were not associated in a Taiwanese AS population [27].

Rheumatoid arthritis (RA) is a type of autoimmune arthritis, triggered by a faulty immune system resulting in chronic synovitis and the destruction of localized cartilage and bone [28]. Previous twin and family-base studies indicate that the associations with major histocompatibility complex (MHC) regions explain around 12% of total heritability in the susceptibility of RA [29]. A recent GWAS meta-analysis indicated that nine new loci were associated with RA in a Japanese population [30]. Because AS and RA are autoimmune diseases that may share similar genetic factors as well as immune regulatory pathways, we therefore examined if the RA susceptibility polymorphisms from GWAS meta-analysis are also associated with the pathogenesis of AS. In this study, we selected eight single-nucleotide polymorphisms (SNPs) from previous GWAS meta-analysis study. The AS activity index (Bath AS Disease Activity Index, BASDAI), Bath AS Functional Index (BASFI), and Bath AS Global (BAS-G), as well as inflammatory biochemical parameters (immunoglobulin A, IgA, erythrocyte sedimentation rate, ESR, and CRP) were analyzed. We found that the AA genotype of rs657075 (*CSF2*) was significantly associated to the clinical phenotype Bath AS Global (BAS-G).

## 2. Results

### 2.1. Association Study between RA Polymorphisms and Susceptibility to AS

Eight selected RA polymorphisms from previous GWAS meta-analysis were selected for genotyping (Table 1). A total of 475 AS patients were recruited in this study (Table 2). The mean age was 39.1 ± 11.3 years; 68% were men, and 431 patients (90.7%) were *HLA-B27* (+). Their mean BASDAI, BASFI and BAS-G scores were 4.3 ± 2.2, 2.1 ± 2.2, and 4.4 ± 2.8, respectively. The genotypes of 11,301 healthy subjects were used as controls to analyze the susceptibility to AS. All genotyped SNPs were in Hardy–Weinberg equilibrium (Table S1). As shown in Table 3, no significant association was found between SNPs and the susceptibility of AS. Two SNPs, rs11900673 (*B3GNT2*) and rs2847297 (*PTPN2*), showed an significant association with *HLA-B27*-positive AS patients in the recessive model (*p* = 0.0394) and dominant model (*p* = 0.0316), respectively (Table S2). However, the significance disappeared after the Bonferroni correction.

### 2.2. rs657075 Is Associated with the Disease Activity Index

We further analyzed the correlation between genetic polymorphisms and clinical phenotypes including the BASDAI, BASFI and BAS-G in the AS patients (Table 4). The results indicated that a higher score of BAS-G in AS patients was associated with the AA genotypes of rs657075 (*CSF2*) (*p* = 0.011; *q* = 0.088). Subgroup analysis indicated a similar result (*p* = 0.021; *q* = 0.168) in *HLA-B27*-positive AS patients (Table S3). However, no association was found between these genetic polymorphisms and other two phenotypes.

### 2.3. Association Study between RA Polymorphisms and Inflammatory Biochemical Parameters of AS

The correlation of genetic polymorphisms with inflammatory biochemical parameters (IgA, ESR and CRP) in AS patients were also evaluated (Table 5). The rs657075 (*CSF2*) AA genotype was associated with increased level of IgA (*p* = 0.029), however this is insignificant after multiple testing correction (*q* = 0.194). Subgroup analysis indicated a weak correlation between rs11900673 (*B3GNT2*) and the ESR level in *HLA-B27*-positive patients, but the result was not still significant after multiple testing correction (*p* = 0.029; *q* = 0.232) (Table S4).

### 2.4. Studies for Tissue Expression Quantitative Trait Loci (eQTLs) of rs657075

**Figure 1:**
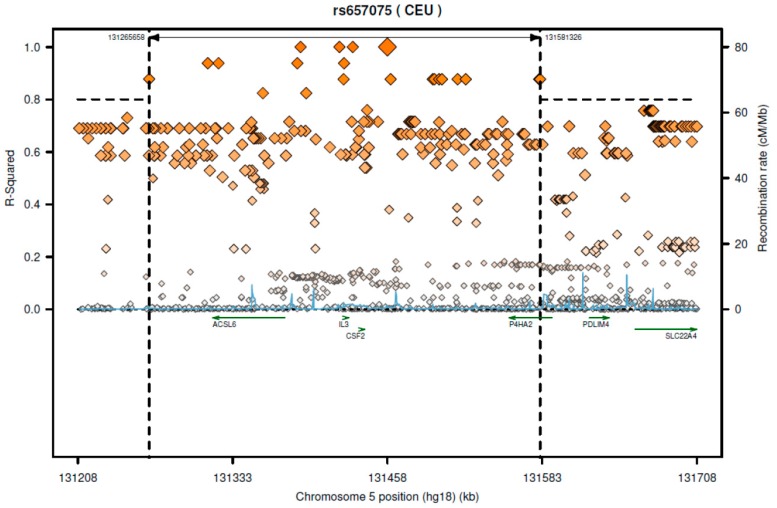
The regional LD plot of rs657075 in CEU population.

Based on 1000-genome CEU (Utah residents with Northern and Western European ancestry from the CEPH collection) population, this SNP is in great LD (linkage disequilibrium) (*r*^2^ > 0.8) with several SNPs which are located on different genes such as *CSF2*, acyl-CoA synthetase long-chain family member 6 (*ACSL6*), *IL3*, and *P4HA2* (Figure 1). From data of the GTEx portal, we found that rs657075 can influence the expression of *ACSL6* gene in colon-sigmoid cells (*p* = 4.7 × 10^−7^) (Table 6).

## 3. Discussion

AS and RA are autoimmune diseases with distinct phenotypes. RA is characterized as a chronic inflammatory joint disease with cartilage and bone damage, whereas spondyloarthropathies in AS are illustrated by entheses and subchondral bone marrow inflammation, particularly abnormal osteoproliferation at involved sites. Familial aggregations of AS and RA have been known for years [31,32]. In a 2008 Sweden study, Sundquist and coworkers analyzed 30 years of hospitalizations (1973–2004) for concordant and discordant associations among RA, AS, and systemic lupus erythematosus (SLE). They reported that significant concordant measures with standardized incidence ratios (SIRs) or sibling risks for RA and AS were 5.12 and 17.14, respectively. However, the discordant association between RA and AS was not significant, and AS was only associated with AS when discordance was taken into account [31]. A further study in 2009 indicated that offspring of parental probands with RA were significantly associated with AS with a SIR of 2.96 [32]. This laid the foundation for the identification of common genetic components for RA and AS. Recently, some autoimmune disease loci were identified as being shared among multiple autoimmune diseases [33,34]. Sirota and coworkers (2009) compared genetic variation profiles of six autoimmune disease and found that RA and AS displayed an autoimmune disease locus cluster that was distinct from the others. For instance, the G allele of rs2076530 in *BTNL2* predisposed patients to RA, AS, and type 1 diabetes, but may play a protective role instead in multiple sclerosis and autoimmune thyroid disease.

To date, more than 43 AS risky loci [24] and 101 RA risky loci [35] have been identified. Most of the identified risk loci for autoimmune diseases are related to B-cell or T-cell activation pathways, differentiation, or innate immunity, or involved cytokine signaling or regulating peripheral tolerance. *HLA-B27* has been known for years to be the major AS-susceptibility gene [6], but fewer than 5% of *HLA-B27* carriers develop AS [24], suggesting that non-*HLA-B27* alleles are important for AS susceptibility. Indeed, we recently provided evidence that genetic polymorphisms of *ORAI1* (rs12313273 and rs7135617) and *STIM1* (rs3750996) were associated with the pathogenesis of *HLA-B27*-positive AS patients [18,19]. There is evidence that three of the SNPs (rs4552569, rs17095830, and rs13210693) which correspond to two newly identified AS risky loci (*EDIL3*-*HAPLN1* and *ANO6*) in Han Chinese [22] were not replicated in Caucasian populations [20,21,23] and were negatively associated with Taiwanese AS subjects in a previous study of ours [27], eliciting the question of whether these two newly-identified loci associated with AS in Han Chinese [22] may have been over-represented. Indeed the association of one SNP, rs13210693 at the 6q21 locus, did not reach a *p* value at the genome-wide significance level (*p* > 5 × 10^−8^) [22].

Bath ankylosing spondylitis global score (BAS-G) was a valuable quantitative measure which gives a global assessment of the well-being of the person with AS over a given time period as completed by the patient. It was first reported by Jones et al. in 1960 [36], and endorsed by Assessment of SpondyloArthritis international Society (ASAS) [37]. It uses two horizontal visual analogue scales (10 cm) to measure the effect of AS on the respondent’s well-being, where none = 0 and severe = 10. The first estimates over the last week, and the second over the last six months. In this study, we firstly reported that the genetic polymorphism rs657075 (*CSF2*) was associated with BAS-G.

*CSF2* loci were previously reported to be associated with RA and exhibited high levels in joints of RA patients [30]. In this study, for the first time we showed that *CSF2* loci were not associated with AS development, but may be weakly associated with AS clinical phenotype BAS-G levels in a Taiwanese population. *CSF2*, also known as granulocyte-macrophage colony-stimulating factor, functions as a cytokine and is secreted by macrophages, T cells, mast cells, natural killer (NK) cells, endothelial cells, and fibroblasts. It stimulates stem cells to produce granulocytes and is part of the immune/inflammatory cascade. On the other hand, the T helper Th17 cells require *Csf2* to induce autoimmune encephalomyelitis or autoimmune neuroinflammation [38]. It is known that IL-23 and IL-23R are involved in pathogenic Th17 responses, and *IL-23R* is associated with many autoimmune diseases such as psoriasis, AS, and Crohn’s disease [24]. Perhaps levels of the weakly associated AS clinical phenotype BAS-G seen may have something to do with some of these gene–gene interactions.

According to SNAP website, this SNP has high LD with several genes including *CSF2* and *ACSL6* (Figure 1). Since the GTEx database includes limited cell types, rs657075 does not show significantly associated eQTL with the *CSF2* gene in the current database. It is associated with expression of *ACSL6* in sigmoid colon cells (Table 6). On searching HaploReg V4.1, rs657075 is involved in changing chromatin status in primary T cells. Therefore, further functional studies are needed to validate the impact of rs657075 on the expression of *CSF2*.

Using a high-density immune-related loci platform (Immunochip), Cortes and coworkers reported that some of the AS risky loci overlapped with those of other immune diseases. Eleven of these AS risky loci were positively associated with ulcerative colitis and 12 loci with Crohn’s disease. However, only two of the AS risky loci were marginally associated with RA; rs11065898 (*SH2B3* or *LNK*) and rs2283790 (*UBE2L3*) displayed a concordant and discordant mode, respectively. As *SH2B3* encoded an adaptor protein involving T cell signaling, it may be functionally related to the pathogenesis of AS. Interestingly, rs11065898 loci were indeed positively associated with CD4^+^ lymphocyte counts [24].

The modest correlation between RA risk SNPs and AS susceptibility that we observed in this study may due to several reasons.

Firstly, the SNPs were identified from a RA GWAS study conducted in a Japanese cohort. In this study, we assess their contribution to AS susceptibility and disease severity in a Taiwanese cohort. The nonsignificant results regarding susceptibility to Taiwanese AS patients may be attributed to the heterogeneity of the phenotype (diagnostic criteria of the disease). In considering of phenotypic heterogeneity, we have noted different criteria of clinical diagnosis between RA (in the GWAS study from a Japanese population) and AS (in this study based on a Taiwanese population).

Secondly, it is likely that different genetic backgrounds (variation in allele frequencies across different ethnic population admixture) in two populations may play a role in the observed inconsistent results. In particular, different genetic backgrounds between Japanese and Taiwanese populations may have an impact on the genetic influence of rs657075 in the two study populations, consequently, this may result in the inconsistent association that we observed in these two studies.

Thirdly, the prevalences of AS between Taiwanese and Japanese populations are different. The prevalence in Japanese population is lower than in Taiwanese population (0.0065% vs. 0.19%–0.54%) [39,40,41]. The distinct prevalence of AS may imply various genetic factors influencing different underlying regulations in AS across population, which may partially explain the nonsignificant association observed in our study. For example, a previous study reported that three SNPs (rs4552569, rs17095830, and rs13210693) were associated with AS in Han Chinese [22], but this was not replicated in Caucasian population [20,21,23], nor were these associated with Taiwanese AS subjects [27].

Fourthly, it has been known that the genetic effect size of SNP may be different across populations. Since some important confounding factors are unmeasured and can not be adjusted in the analyses, this may have a certain degree of influence when determining the risk effect of those SNPs on AS.

Although no correlation between RA susceptible SNPs and AS risk was observed, we have shown that these SNPs are good candidates for AS disease severity, i.e., BASDAI, BASFI, and BAS-G after adjusting for the effects of gender, age, and disease duration. It would still be interesting to consider those previously identified SNPs as good candidates for determining increased risk of AS. As such, further investigation would be merited to validate these SNPs identified from the Japanese AS GWAS study. It will be also important to the authors to further examine the association between these SNPs and increasing AS risk when sample size allows in the future.

The small sample size in this study may have limited the statistical power for detecting sizeable changes in AS risk assessments. Larger cohort studies and replication studies in different populations are required to validate our findings in this study. In summary, although the SNPs (rs657075 on *CSF2* and rs11900673 on *B3GNT2*) are not the risk alleles for the susceptibility of AS, we provide supportive evidence that these SNPs are important generic markers for clinical manifestations of AS patients in a Taiwanese population. Further investigation would be needed to validate the findings in this study.

## 4. Materials and Methods

### 4.1. Study Subjects

Patients were sequentially recruited from the AS clinic at Chung Shan Medical University Hospital (Taichung, Taiwan). Our study conformed to the *Declaration of Helsinki*, and the design of the work and final report were performed with the approval of the institutional review board of Chung Shan Medical University Hospital (CSMUH No: CS12255). We received informed consent from all subjects before any data were collected. Patients with AS who met the selection criteria were asked to participate in the study. In total 475 AS patients were recruited from the Arthritis Clinic, and diagnosed by a qualified rheumatologist. The criteria included (i) patients being aged 17–82 years; (ii) an AS diagnosis having been made by modified New York criteria developed in 1984 [42]; (iii) being fluent Chinese language speakers; and (iv) having cognitive performance that was not influenced by other disease such as dementia. Patients with sacroiliitis were confirmed by a qualified radiologist. The detailed clinical history included age at initial symptoms, extra-spinal manifestations , and laboratory parameters of inflammation, i.e., the ESR, CRP, and IgA. The age of onset of AS symptom was defined as the time when the first symptom (axial symptom, peripheral arthritis, uveitis, or enthesitis) was noted. The BASDAI, BASFI, and BAS-G were applied to evaluate the disease activity, physical functions, and global wellbeing, respectively. A family history of AS was also recorded. Peripheral arthritis was defined as the presence of at least one swollen joint. These symptoms were determined by a rheumatologist, ophthalmologist, and gastroenterologist.

### 4.2. Candidate Single-Nucleotide Polymorphisms (SNPs)

We included eight SNPs which showed a significant association with RA in previous GWASs in a Japanese population (Table 1) [30]. rs2867461 (*ANXA3*) was excluded from the statistical analysis, because many subjects were unsuccessfully genotyped due to failure of polymerase chain reaction (PCR) amplification.

### 4.3. DNA Extraction

Peripheral venous blood was collected during medical surveillance, and processed in the same day. Blood was centrifuged to separate the serum and cells. DNA of blood cells was extracted by first treating them with 0.5% sodium dodecylsulfate lysis buffer and then protease K (1 mg/mL) to digest nuclear proteins for 4 h at 60 °C according to the manufacturer’s procedures. Total DNA was harvested using a Gentra (Qiagen, Valencia, CA, USA) extraction kit followed by 70% alcohol precipitation as recommended.

### 4.4. Genotyping

Genotyping for the eight SNPs was performed using the TaqMan Allelic Discrimination Assay (Applied Biosystems, Foster City, CA, USA). PCR was performed in the 96-well microplate and with the ABI9700 Thermal Cycler (Applied Biosystems). After the PCR, fluorescence was measured and analyzed using the StepOne software vers. 2.2.2 (Applied Biosystems, Foster City, CA, USA). *HLA-B27* carriage was assessed by flow cytometry as previously described [43].

### 4.5. SNP Annotation Data Query

In order to understand to potential regulated genes by each SNP, we queried SNAP (https://www.broadinstitute.org/mpg/snap/index.php) to download the regional LD plot of interests for SNPs. The GTEx Portal (http://www.gtexportal.org/home/), which includes a variety of tissue expression quantitative trait loci (eQTLs), was used to investigate the association between gene expression profiles and the SNPs.

### 4.6. Statistical Analysis

SAS 9.3 (SAS Institute, Cary, NC, USA) for Windows was used for the statistical analysis. Hardy–Weinberg equilibrium (HWE) was evaluated to test the allelic frequencies of SNP’s using a chi-squared test (χ^2^ test). Statistical differences between the patient and control group in genotype frequencies were evaluated by a χ^2^ test. An analysis of variance (ANOVA) was used to compare the mean of continuous variables (BASDAI, BASFI, BAS-F, IgA, ESR, and CRP) among different genotypes in AS patients. An analysis of covariance (ANCOVA) was applied to adjust for gender, age, and disease duration. The false discovery rate (FDR) was applied in order to correct for multiple testing errors. FDR *q* values of <0.05 was considered statistically significant.

## Figures and Tables

**Table 1:** Eight previously reported single-nucleotide polymorphisms (SNP) associated with rheumatoid arthritis in the Japanese population.

SNP	Chromosome	Position (bp)	Cytoband	Candidate Gene	Context	*p* Value
rs11900673	2	62306165	2p15	*B3GNT2*	NA	1.1 × 10^−^^8^
rs657075	5	131458017	5q31	*CSF2*	NA	2.8 × 10^−^^10^
rs12529514	6	14204637	6p23	*CD83*	NA	2.0 × 10^−^^8^
rs2233434	6	44340898	6p21.1	*NFKBIE*	Exon (Val/Ala)	5.8 × 10^−^^19^
rs10821944	10	63455095	10q21	*ARID5B*	Intron	5.5 × 10^−^^18^
rs3781913	11	72051144	11q13	*PDE2A-ARAP1*	Intron	5.8 × 10^−^^10^
rs2841277	14	104462050	14q32	*PLD4*	Near Gene-5‘	1.9 × 10^−^^14^
rs2847297	18	12787694	18p11	*PTPN2*	Intron	2.2 × 10^−^^8^

These results were extracted from reference [30]. NA, not available.

**Table 2:** Basal characteristics of patients with ankylosing spondylitis (AS) and of Taiwanese biobank controls.

Characteristics	Patients with AS	Taiwanese Biobank Controls
Number of subjects	475	11,301
Gender: Male (%)	323 (68.0%)	
Age (years)	39.1 ± 11.3 ^a^	
Range	17~82	
*HLA-B27^+^*	431 (90.7%)	
BASDAI	4.3 ± 2.2	
BASFI	2.1 ± 2.2	
BAS-G	4.4 ± 2.8	

^a^ Mean ± S.D. BASDAI, Bath AS Disease Activity Index; BASFI, Bath AS Functional Index; BAS-G, Bath AS Global, HLA: human leucocyte antigen.

**Table 3:** Genotype and allelic frequencies in a Taiwanese biobank of controls and patients with ankylosing spondylitis.

Single-Nucleotide Polymorphism	Genotype	Cases (%) (*n* = 475)	Control Subjects (%) (*n* = 11,301)	Allele	Cases (%) (*n* = 475)	Control Subjects (%) (*n* = 11,301)	Dominant *p* Value	Recessive *p* Value	Allelic *p* Value
*B3GNT2*	TT	5 (1.3)	346 (3.0)	T	118 (15.6)	4008 (17.7)	0.2993	0.0512	0.1191
rs11900673	CT	108 (28.6)	3316 (29.4)	C	638 (84.4)	18,574 (82.3)			
	CC	265 (70.1)	7629 (67.6)						
*CSF2*	AA	27 (7.1)	711 (6.3)	A	185 (24.2)	5715 (25.7)	0.2272	0.5558	0.4579
rs657075	GA	131 (34.2)	4293 (38.1)	G	581 (75.8)	16,481 (74.3)			
	GG	225 (58.7)	6274 (55.6)						
*CD83*	CC	28 (7.1)	613 (5.4)	C	199 (25.3)	5319 (23.6)	0.5013	0.1514	0.2731
rs12529514	TC	143 (36.3)	4093 (36.3)	T	589 (74.7)	17,251 (76.5)			
	TT	223 (56.6)	6579 (58.3)						
*NFKBIE*	CC	11 (2.5)	12 (2.6)	C	135 (15.1)	151 (16.6)	0.3533	0.8665	0.3855
rs2233434	TC	113 (25.3)	127 (28.0)	T	757 (84.9)	757 (83.4)			
	TT	322 (72.2)	315 (69.4)						
*ARID5B*	GG	32 (7.9)	792 (7.0)	G	209 (25.7)	5988 (26.5)	0.3120	0.5123	0.5901
rs10821944	TG	145 (35.6)	4404 (39.0)	T	605 (74.3)	16,588 (73.3)			
	TT	230 (56.5)	6092 (54.0)						
*PDE2A-ARAP1*	CC	47 (10.8)	44 (8.9)	C	275 (31.5)	284 (28.6)	0.2362	0.3280	0.1731
rs3781913	AC	181 (41.4)	196 (39.4)	A	599 (68.5)	710 (74.3)			
	AA	209 (47.8)	257 (51.7)						
*PLD4*	CC	82 (18.3)	1954 (17.3)	C	379 (42.4)	9370 (41.5)	0.7506	0.5730	0.6012
rs2841277	TC	215 (48.1)	5462 (48.4)	T	515 (57.6)	13,200 (58.5)			
	TT	150 (33.6)	3869 (34.3)						
*PTPN2*	GG	40 (9.3)	1053 (9.2)	G	273 (31.7)	6728 (29.8)	0.1447	0.9848	0.2640
rs2847297	AG	193 (44.8)	4658 (41.2)	A	589 (68.3)	15,862 (70.2)			
	AA	198 (45.9)	5602 (49.6)						

**Table 4:** Difference in the scores of the Bath Ankylosing Spondylosis (AS) Disease Activity Index (BASDAI), Bath AS Functional Index (BASFI), and Bath AS Global (BAS-G) among AS patients stratified by different genotypes.

Single-Nucleotide Polymorphism	Genotype	BASDAI	BASFI	BAS-G
*B3GNT2*	TT	3.1 ± 0.9 ^a^	0.8 ± 0.8	3.9 ± 3.3
rs11900673	CT	4.3 ± 2.3	1.9 ± 2.1	4.3 ± 2.7
	CC	4.3 ± 2.1	2.1 ± 2.3	4.4 ± 2.7
*p* value ^†^		0.322	0.262	0.834
*q* value		0.858	0.699	0.886
*CSF2*	AA	5.1 ± 2.1	2.4 ± 2.2	6.1 ± 2.8
rs657075	GA	4.3 ± 2.3	2.0 ± 2.2	4.4 ± 2.7
	GG	4.3 ± 2.2	2.0 ± 2.3	4.2 ± 2.8
*p* value ^†^		0.452	0.816	0.011
*q* value		0.858	0.848	0.088
*CD83*	CC	4.9 ± 2.2	2.2 ± 2.0	5.1 ± 2.9
rs12529514	TC	4.3 ± 2.2	2.1 ± 2.3	4.5 ± 2.7
	TT	4.4 ± 2.2	2.1 ± 2.2	4.3 ± 2.7
*p* value ^†^		0.772	0.848	0.528
*q* value		0.858	0.848	0.780
*NFKBIE*	CC	4.9 ± 1.8	1.7 ± 1.7	3.5 ± 2.1
rs2233434	TC	4.4 ± 2.2	2.0 ± 2.2	4.8 ± 2.7
	TT	4.3 ± 2.2	2.1 ± 2.3	4.3 ± 2.8
*p* value ^†^		0.725	0.759	0.137
*q* value		0.858	0.848	0.403
*ARID5B*	GG	4.3 ± 2.1 ^a^	2.2 ± 2.0	4.6 ± 3.1
rs10821944	TG	4.5 ± 2.2	2.2 ± 2.3	4.6 ± 2.7
	TT	4.3 ± 2.2	2.0 ± 2.2	4.3 ± 2.7
*p* value ^†^		0.799	0.674	0.483
*q* value		0.858	0.848	0.780
*PDE2A-ARAP1*	CC	3.9 ± 2.3	1.7 ± 2.0	4.0 ± 2.8
rs3781913	AC	4.3 ± 2.2	2.0 ± 2.3	4.4 ± 2.8
	AA	4.5 ± 2.2	2.2 ± 2.2	4.5 ± 2.8
*p* value ^†^		0.239	0.198	0.585
*q* value		0.858	0.699	0.780
*PLD4*	CC	4.3 ± 2.3	1.8 ± 1.8	4.3 ± 2.6
rs2841277	TC	4.4 ± 2.1	2.2 ± 2.3	4.3 ± 2.7
	TT	4.3 ± 2.2	2.0 ± 2.3	4.6 ± 3.0
*p* value ^†^		0.858	0.218	0.886
*q* value		0.858	0.699	0.886
*PTPN2*	GG	4.7 ± 2.6	2.3 ± 2.6	4.8 ± 3.1
rs2847297	AG	4.2 ± 2.2	2.0 ± 2.2	4.2 ± 2.7
	AA	4.5 ± 2.1	2.1 ± 2.2	4.6 ± 2.7
*p* value ^†^		0.405	0.603	0.151
*q* value		0.858	0.848	0.403

^a^ Data are presented as the mean ± SD; ^†^ Adjusted for the effects of age, sex, and disease duration.

**Table 5:** Differences in the values of immunoglobulin A (IgA), the erythrocyte sedimentation rate (ESR), and C-reactive protein (CRP) among ankylosing spondylitis (AS) patients stratified by different genotypes.

Single-Nucleotide Polymorphism	Genotype	IgA (mg/dL)	ESR (mm/h)	CRP (mg/dL)
*B3GNT2*	TT	263.40 ± 53.86 ^a^	11.60 ± 11.63	0.49 ± 0.27
rs11900673	CT	307.39 ± 113.43	22.15 ± 18.14	0.93 ± 1.67
	CC	317.30 ± 125.25	25.97 ± 20.93	1.22 ± 1.91
*p* value ^†^		0.500	0.065	0.180
*q* value		0.571	0.520	0.720
*CSF2*	AA	371.31 ± 151.17	26.59 ± 20.15	1.52 ± 1.75
rs657075	GA	304.23 ± 121.55	25.64 ± 22.61	1.17 ± 1.93
	GG	322.66 ± 119.54	23.02 ± 17.98	0.98 ± 1.58
*p* value ^†^		0.029	0.541	0.274
*q* value		0.194	0.845	0.731
*CD83*	CC	351.13 ± 163.63	25.57 ± 25.22	1.31 ± 1.98
rs12529514	TC	330.24 ± 130.04	25.58 ± 18.73	1.08 ± 1.63
	TT	312.55 ± 116.45	25.17 ± 22.05	1.20 ± 1.96
*p* value ^†^		0.087	0.891	0.827
*q* value		0.194	0.845	0.945
*NFKBIE*	CC	301.55 ± 116.79	35.30 ± 24.70	1.76 ± 2.16
rs2233434	TC	301.49 ± 112.85	24.60 ± 22.42	1.16 ± 1.70
	TT	330.32 ± 132.87	25.38 ± 20.66	1.15 ± 1.88
*p* value ^†^		0.176	0.484	0.594
*q* value		0.235	0.845	0.945
*ARID5B*	GG	369.07 ± 167.58	27.03 ± 25.29	1.33 ± 1.81
rs10821944	TG	324.63 ± 129.37	25.16 ± 20.59	1.18 ± 2.00
	TT	312.27 ± 115.63	25.22 ± 20.84	1.08 ± 1.70
*p* value ^†^		0.109	0.845	0.816
*q* value		0.194	0.845	0.945
*PDE2A-ARAP1*	CC	306.59 ± 137.91	21.81 ± 22.15	1.11 ± 2.12
rs3781913	AC	306.65 ± 107.39	24.65 ± 19.00	1.15 ± 1.82
	AA	336.00 ± 132.63	25.64 ± 21.70	1.15 ± 1.76
*p* value ^†^		0.118	0.651	0.995
*q* value		0.194	0.845	0.995
*PLD4*	CC	325.88 ± 142.40	22.71 ± 18.27	1.08 ± 1.92
rs2841277	TC	310.67 ± 126.94	26.50 ± 23.30	1.15 ± 1.84
	TT	338.13 ± 122.76	23.19 ± 18.32	1.09 ± 1.69
*p* value ^†^		0.121	0.241	0.788
*q* value		0.194	0.845	0.945
*PTPN2*	GG	323.91 ± 140.76	28.00 ± 21.32	1.66 ± 2.55
rs2847297	AG	318.27 ± 129.70	25.58 ± 22.68	1.15 ± 1.68
	AA	322.05 ± 120.44	23.78 ± 18.89	1.03 ± 1.84
*p* value ^†^		0.788	0.451	0.162
*q* value		0.788	0.845	0.720

^a^ Data are presented as the mean ± SD; ^†^ Adjusted for the effects of age, sex, and disease duration.

**Table 6:** Expression Quantitative Trait Loci (eQTL) results from Genotype-Tissue Expression (GTEx).

SNP ID	Gencode ID (ENSG00000-)	Gene Symbol	*p* Value	Effect Size	Tissue	Actions
rs657075	164398.8	*ACSL6*	4.70 × 10^−7^	1.00	Colon, sigmoid	AG > GG
rs3781913	186635.10	*ARAP1*	1.40 × 10^−8^	−0.29	Esophagus, mucosa	TT > GT > GG
	214530.3	*STARD10*	1.40 × 10^−6^	0.38	Heart, atrial appendage	TT < GT < GG
	256007.1	*ARAP1-AS1*	3.00× 10^−6^	−0.18	Esophagus, mucosa	TT > GT > GG
	214530.3	*STARD10*	3.20 × 10^−6^	0.19	Whole blood	TT < GT < GG
	214530.3	*STARD10*	3.30 × 10^−6^	0.20	Cells, transformed fibroblasts	TT < GT < GG
	214530.3	*STARD10*	1.00 × 10^−5^	0.16	Muscle, skeletal	TT < GT < GG
rs2841277	140104.9	*C14orf79*	1.10 × 10^−8^	0.29	Cells, transformed fibroblasts	CC < CT < TT
	140104.9	*C14orf79*	2.10 × 10^−8^	0.44	Brain, cortex	CC < CT < TT
	140104.9	*C14orf79*	6.60 × 10^−7^	0.25	Skin, sun-exposed (lower leg)	CC < CT < TT
	140104.9	*C14orf79*	2.30 × 10^−6^	0.47	Brain, cerebellum	CC < CT < TT
	140104.9	*C14orf79*	8.30 × 10^−6^	0.29	Esophagus, muscularis	CC < CT < TT
rs2847297	267654.1	*RP11-973H7.4*	6.60 × 10^−14^	0.45	Whole blood	AA < AG < GG
	260302.1	*RP11-973H7.1*	2.10 × 10^−10^	0.49	Artery, tibial	AA < AG < GG
	260302.1	*RP11-973H7.1*	7.20 × 10^−9^	0.52	Artery, aorta	AA < AG < GG
	260302.1	*RP11-973H7.1*	2.70 × 10^−7^	0.48	Esophagus, muscularis	AA < AG < GG
	260302.1	*RP11-973H7.1*	4.10 × 10^−7^	0.40	Lung	AA < AG < GG
	260302.1	*RP11-973H7.1*	5.00 × 10^−7^	0.25	Whole blood	AA < AG < GG

## Supplementary Materials

Supplementary materials can be found at www.mdpi.com/1422-0067/18/1/83/s1.
