# Supplementary material for: rs657075 (CSF2) Is Associated with the Disease Phenotype (BAS-G) of Ankylosing Spondylitis
Source: Int J Mol Sci. 2017 Jan 3;18(1):83. doi: 10.3390/ijms18010083 (PMC5297717; doi:10.3390/ijms18010083)
Supplement: Supplementary file 1 [file ijms-18-00083-s001.pdf]

Supplementary Materials: rs657075 (*CSF2*) Associated with the Disease Phenotype (BAS-G) of Ankylosing Spondylitis

Wei-Chiao Chen, James Cheng-Chung Wei, Hsing-Fang Lu, Henry Sung-Ching Wong, Peng Yeong Woon, Yu-Wen Hsu, Jin-Ding Huang and Wei-Chiao Chang

Table S1. The basic characteristics of the SNPs.

Gene	Pos (hg38)	Variant	Ref	Alt	AFR Freq	AMR Freq	ASN Freq	EUR Freq	JPT Freq	TWB Freq	HWE
B3GNT2	chr2:62225526	rs11900673	C	T	0.12	0.23	0.24	0.13	0.28	0.18	0.53
CSF2	chr5:132094425	rs657075	G	A	0.02	0.07	0.31	0.13	0.36	0.26	0.52
CD83	chr6:14096427	rs12529514	T	C	0.01	0.03	0.19	0.06	0.14	0.23	0.47
NFKBIE	chr6:44265183	rs2233434	A	G	0.05	0.02	0.16	0.04	0.21	0.16	0.85
ARID5B	chr10:62025330	rs10821944	G	T	0.57	0.64	0.70	0.69	0.64	0.74	0.92
PDE2A-ARAP1	chr11:72662452	rs3781913	T	G	0.59	0.48	0.28	0.54	0.31	0.28	0.45
PLD4	chr14:104924668	rs2841277	C	T	0.22	0.45	0.66	0.47	0.69	0.58	0.73
PTPN2	chr18:12797695	rs2847297	A	G	0.57	0.27	0.34	0.32	0.33	0.30	0.06

Freq frequency shows as alt allele. TWB frequency based on Taiwan Biobank. HWE p value for Hardy-Weinberg equilibrium.

Table S2. Genotype and allelic frequencies in a Taiwanese biobank of controls and patients with HLA-B27 (+) ankylosing spondylitis.

Single-Nucleotide Polymorphism	Genotype	Cases (%) (*n* = 431)	Control Subjects (%) (*n* = 11,301)	Allele	Cases (%) (*n* = 431)	Control Subjects (%) (*n* = 11,301)	Dominant *p* Value	Recessive *p* Value	Allelic *p* Value
B3GNT2	TT	4 (1.2)	346 (3.0)	T	107 (15.4)	4008 (17.7)	0.2655	**0.0394** *	0.1057
rs11900673	CT	99 (28.4)	3316 (29.4)	C	589 (84.6)	18,574 (82.3)			
	CC	245 (70.4)	7629 (67.6)						
CSF2	AA	22 (6.3)	711 (6.3)	A	165 (23.6)	5715 (25.7)	0.2085	0.9996	0.3094
rs657075	GA	121 (34.7)	4293 (38.1)	G	533 (76.4)	16,481 (74.3)			
	GG	206 (59.0)	6274 (55.6)						
CD83	CC	25 (6.9)	613 (5.4)	C	179 (24.7)	5319 (23.6)	0.7497	0.2250	0.4705
rs12529514	TC	129 (35.6)	4093 (36.3)	T	545 (75.3)	17,251 (76.5)			
	TT	208 (57.5)	6579 (58.3)						
NFKBIE	CC	10 (2.5)	12 (2.6)	C	127 (15.6)	151 (16.6)	0.5646	0.8674	0.5778
rs2233434	TC	107 (26.3)	127 (28.0)	T	685 (84.4)	757 (83.4)			
	TT	289 (71.2)	315 (69.4)						
ARID5B	GG	28 (7.9)	792 (7.0)	G	189 (25.5)	5988 (26.5)	0.3163	0.6940	0.5228
rs10821944	TG	133 (35.6)	4404 (39.0)	T	553 (74.5)	16,588 (73.3)			
	TT	210 (56.5)	6092 (54.0)						
PDE2A-ARAP1	CC	41 (10.3)	44 (8.9)	C	244 (30.7)	284 (28.6)	0.2362	0.3280	0.1731
rs3781913	AC	162 (40.8)	196 (39.4)	A	550 (69.3)	710 (74.3)			
	AA	194 (48.9)	257 (51.7)						
PLD4	CC	76 (18.6)	1954 (17.3)	C	344 (42.2)	9370 (41.5)	0.9902	0.4917	0.7148
rs2841277	TC	192 (47.1)	5462 (48.4)	T	472 (57.8)	13,200 (58.5)			
	TT	140 (34.3)	3869 (34.3)						
PTPN2	GG	38 (9.7)	1053 (9.2)	G	257 (32.9)	6728 (29.8)	**0.0316** *	0.7836	0.0748
rs2847297	AG	181 (46.3)	4658 (41.2)	A	525 (67.1)	15,862 (70.2)			
	AA	172 (44.0)	5602 (49.6)						

* Significant ($p < 0.05$) values are in bold.

Table S3. Differences in scores of the Bath Ankylosing Spondylosis (AS) Disease Activity Index (BASDAI), Bath AS Functional Index (BASFI), and Bath AS Global (BAS-G) among HLA-B27 (+) AS patients stratified by different genotypes.

Single-Nucleotide Polymorphism	Genotype	BASDAI	BASFI	BAS-G
B3GNT2	TT	3.3 ± 0.9 [a]	0.7 ± 0.9	3.0 ± 3.1
rs11900673	CT	4.3 ± 2.2	1.9 ± 2.2	4.4 ± 2.6
	CC	4.3 ± 2.1	2.1 ± 2.3	4.3 ± 2.7
p value [†]		0.539	0.269	0.354
q value		0.933	0.858	0.641
CSF2	AA	5.0 ± 2.2	2.4 ± 2.2	6.2 ± 2.8
rs657075	GA	4.3 ± 2.3	2.1 ± 2.3	4.5 ± 2.7
	GG	4.2 ± 2.2	2.0 ± 2.2	4.2 ± 2.7
p value [†]		0.574	0.821	0.021
q value		0.933	0.858	0.168
CD83	CC	5.0 ± 2.2	2.3 ± 2.1	5.3 ± 3.0
rs12529514	TC	4.2 ± 2.2	2.0 ± 2.3	4.5 ± 2.7
	TT	4.4 ± 2.1	2.1 ± 2.2	4.3 ± 2.7
p value [†]		0.583	0.858	0.401
q value		0.933	0.858	0.641
NFKBIE	CC	4.7 ± 1.9	1.6 ± 1.7	3.6 ± 2.2
rs2233434	TC	4.3 ± 2.2	2.0 ± 2.1	4.7 ± 2.7
	TT	4.3 ± 2.1	2.1 ± 2.3	4.3 ± 2.8
p value [†]		0.760	0.669	0.262
q value		0.987	0.858	0.641

Table S3. *Cont.*

Single-Nucleotide Polymorphism	Genotype	BASDAI	BASFI	BAS-G
ARID5B	GG	4.3 ± 2.2 [a]	2.3 ± 2.0	4.7 ± 3.1
rs10821944	TG	4.4 ± 2.2	2.1 ± 2.3	4.5 ± 2.7
	TT	4.3 ± 2.2	2.0 ± 2.3	4.3 ± 2.7
p value [†]		0.906	0.695	0.481
q value		0.987	0.858	0.641
PDE2A-ARAP1	CC	3.9 ± 2.3	1.7 ± 2.0	4.2 ± 2.8
rs3781913	AC	4.3 ± 2.2	2.0 ± 2.3	4.4 ± 2.7
	AA	4.4 ± 2.2	2.2 ± 2.2	4.5 ± 2.8
p value [†]		0.576	0.500	0.908
q value		0.933	0.858	0.917
PLD4	CC	4.2 ± 2.2	1.7 ± 1.7	4.2 ± 2.5
rs2841277	TC	4.3 ± 2.1	2.2 ± 2.3	4.4 ± 2.7
	TT	4.3 ± 2.3	2.0 ± 2.3	4.5 ± 3.0
p value [†]		0.987	0.192	0.917
q value		0.987	0.858	0.917
PTPN2	GG	4.6 ± 2.5	2.2 ± 2.5	4.6 ± 3.1
rs2847297	AG	4.2 ± 2.2	2.0 ± 2.2	4.2 ± 2.7
	AA	4.4 ± 2.1	2.1 ± 2.3	4.6 ± 2.7
p value [†]		0.534	0.565	0.378
q value		0.933	0.858	0.641

[a] Data are presented as the mean ± SD. [†] Adjusted for the effects of age, sex, and disease duration.

Table S4. Differences in the values of immunoglobulin A (IgA), the erythrocyte sedimentation rate (ESR), and C-reactive protein (CRP) among HLA-B27 (+) ankylosing spondylosis (AS) patients stratified by different genotypes.

Single-Nucleotide Polymorphism	Genotype	IgA (mg/dL)	ESR (mm/h)	CRP (mg/dL)
B3GNT2	TT	261.50 ± 62.00 [a]	6.50 ± 2.65	0.45 ± 0.30
rs11900673	CT	312.89 ± 108.81	21.89 ± 17.93	0.94 ± 1.71
	CC	318.83 ± 121.71	26.48 ± 21.21	1.27 ± 1.97
p value [†]		0.684	0.029	0.178
q value		0.768	0.232	0.784
CSF2	AA	335.14 ± 137.68	22.18 ± 16.65	1.26 ± 1.72
rs657075	GA	307.86 ± 122.59	26.32 ± 23.15	1.23 ± 1.98
	GG	328.20 ± 120.08	23.62 ± 18.12	1.03 ± 1.63
p value [†]		0.486	0.846	0.473
q value		0.648	0.846	0.915
CD83	CC	335.14 ± 137.68	22.18 ± 16.65	1.46 ± 2.05
rs12529514	TC	307.86 ± 122.59	26.32 ± 23.15	1.13 ± 1.68
	TT	328.20 ± 120.08	23.62 ± 18.12	1.21 ± 1.99
p value [†]		0.037	0.553	0.801
q value		0.296	0.726	0.915
NFKBIE	CC	304.00 ± 122.81	33.00 ± 25.03	1.64 ± 2.25
rs2233434	TC	303.59 ± 113.37	24.20 ± 22.33	1.16 ± 1.69
	TT	332.09 ± 130.30	26.00 ± 20.96	1.20 ± 1.95
p value [†]		0.200	0.525	0.645
q value		0.376	0.726	0.915

Table S4. *Cont.*

Single-Nucleotide Polymorphism	Genotype	IgA (mg/dL)	ESR (mm/h)	CRP (mg/dL)
ARID5B	GG	359.88 ± 171.70	29.07 ± 26.34	1.39 ± 1.87
rs10821944	TG	331.17 ± 129.61	25.88 ± 20.91	1.26 ± 2.07
	TT	312.76 ± 112.04	25.01 ± 20.89	1.09 ± 1.74
p value †		0.235	0.635	0.694
q value		0.376	0.726	0.915
PDE2A-ARAP1	CC	304.56 ± 135.02	22.00 ± 23.25	1.21 ± 2.23
rs3781913	AC	308.21 ± 102.06	24.25 ± 18.72	1.17 ± 1.86
	AA	335.52 ± 132.66	26.25 ± 22.00	1.19 ± 1.81
p value †		0.141	0.552	0.983
q value		0.376	0.726	0.983
PLD4	CC	327.62 ± 142.01	22.76 ± 18.40	1.13 ± 1.98
rs2841277	TC	313.44 ± 126.69	27.81 ± 23.83	1.25 ± 1.92
	TT	336.22 ± 118.80	22.45 ± 18.09	1.07 ± 1.71
p value †		0.199	0.078	0.640
q value		0.376	0.312	0.915
PTPN2	GG	325.47 ± 142.57	28.08 ± 21.87	1.70 ± 2.61
rs2847297	AG	319.24 ± 128.04	25.86 ± 22.97	1.20 ± 1.72
	AA	324.53 ± 116.80	24.14 ± 18.88	1.07 ± 1.91
p value †		0.768	0.544	0.196
q value		0.768	0.726	0.784

[a] Data are presented as the mean ± SD. † Adjusted for the effects of age, sex, and disease duration.
